# Semantics

**DOI:** 10.1007/s10912-022-09741-6

**Published:** 2022-06-08

**Authors:** Ien Li

**Affiliations:** David Geffen School of Medicine at UCLA, Los Angeles, CA United States

**Keywords:** burnout, medical student, medical education

Clammy are my hands squirming inside latex gloves,

Picking at zigzag wrinkles

Cascading down ill-fitting scrub pants.

Under trimmed bangs, my wide eyes peer out

Through safety goggles one exhalation away

From a blanket of condensation.

My mask—it obscures both my frozen smile

And the heart in my throat.

Every third of an hour in the geriatric clinic,

A cleaned room seats a new, frail,

Patient in the flesh.

Their hands, gripping sheets of written griefs,

Refrain from shaking mine

As they ready to recount the reasons

They dared break strict isolation

For these precious minutes

With not their loved ones,

But a tremulous trainee.

I should have shriveled from pure unworthiness

When the dainty lady recessed in the corner chair,

With her reading glasses and notebook ready,

Ignored the first half of my introduction as *Student Doctor*

And waved off my stammered correction with,

“You’re a full doctor to me.”

Yet in an instant, my back stood straighter

The light at the end of the tunnel shone brighter

The gut-wrenching, heart-rending,

Learning moments of school nights past

Slackened their fierce grip around my lungs,

And my throat buried deep the primal scream

That so often punctuates twilight-hour studying.

As the two-syllable melody of *doctor*

Swelled my chest

And ricocheted in my eardrums,

I carefully harbored them within every muscle of my being

For the longest of nights and days to come.

## Declarations

This manuscript has not been previously published and is not under consideration in the same or substantially similar form in any other journal.

All those listed as authors are qualified for authorship and all who are qualified to be authors are listed as authors on the byline.

Li, Ien is the sole author responsible for the ideation, writing, and editing of this final submission.

